# Biogenesis, Mode of Action and the Interactions of Plant Non-Coding RNAs

**DOI:** 10.3390/ijms241310664

**Published:** 2023-06-26

**Authors:** Xin Zhang, Mingjun Du, Zhengfu Yang, Zhengjia Wang, Kean-Jin Lim

**Affiliations:** State Key Laboratory of Subtropical Silviculture, College of Forestry and Biotechnology, Zhejiang A&F University, Hangzhou 311300, China; zxx@stu.zafu.edu.cn (X.Z.); 2022602121027@stu.zafu.edu.cn (M.D.); zafuyzf@163.com (Z.Y.)

**Keywords:** non-coding RNA, biogenesis, growth, regulation, stress

## Abstract

The central dogma of genetics, which outlines the flow of genetic information from DNA to RNA to protein, has long been the guiding principle in molecular biology. In fact, more than three-quarters of the RNAs produced by transcription of the plant genome are not translated into proteins, and these RNAs directly serve as non-coding RNAs in the regulation of plant life activities at the molecular level. The breakthroughs in high-throughput transcriptome sequencing technology and the establishment and improvement of non-coding RNA experiments have now led to the discovery and confirmation of the biogenesis, mechanisms, and synergistic effects of non-coding RNAs. These non-coding RNAs are now predicted to play important roles in the regulation of gene expression and responses to stress and evolution. In this review, we focus on the synthesis, and mechanisms of non-coding RNAs, and we discuss their impact on gene regulation in plants.

## 1. Introduction

**Figure 1:**
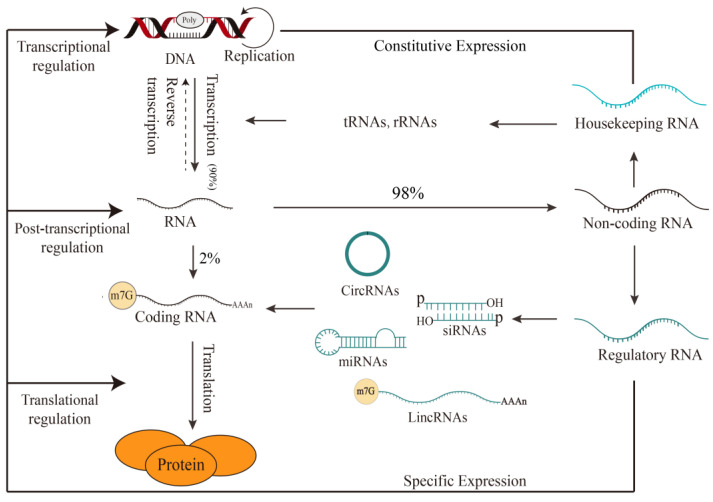
The central dogma and plant non-coding RNAs. As the primary transcription product, plant non-coding RNA transmits genetic information through biological macromolecules and plays a direct role in or outside the cell. Non-coding RNAs range from housekeeping RNAs that differ in expression and function to regulatory RNAs specifically expressed to help plants respond to changes in different habitats. Housekeeping RNAs consist of tRNAs and rRNAs, while regulatory RNAs can be categorized into circular RNA (circRNA), long intergenic non-coding RNA (lincRNA), microRNA (miRNA), and small interfering RNA (siRNA) according to different categories. The arrows represent the direction of biological process. M7G, a modification that a methyl group is added to the N^7^ position of guanine (G) of mRNA.

The current genetic central dogma states that the transmission of genetic information in organisms is multidirectional. Transcription and translation can decode genetic information from DNA to proteins, and the information can also be transferred from RNA to DNA by reverse transcription and replication. Recent studies have pointed out that 98% of the RNAs produced by plants’ DNA transcription are not translated into proteins, which suggests that the genetic information carried by these RNAs is not transmitted in the direction indicated by genetic central dogma [1] (Figure 1). Those RNA molecules which do not encode proteins or have certain biological functions are called non-coding RNAs.

Transfer RNAs (tRNAs) and ribosomal RNAs (rRNAs), which account for many non-coding RNAs, have well-defined functions as translation elements [2]. Therefore, non-coding RNAs can be divided into housekeeping non-coding RNAs, such as constitutively expressed tRNA or rRNA, and non-coding regulatory RNAs that are expressed at specific periods according to their content. The non-coding RNAs featured in this review are non-coding regulatory RNAs.

Non-coding RNAs can be classified into circular RNAs and linear RNAs based on their structure. The length of linear RNAs can be divided into short non-coding RNAs (lengths less than 200 nt), such as microRNA (miRNA), small interfering RNA (siRNA), and long non-coding RNAs (with lengths more than 200 nt) such as long intergenic non-coding RNA (lincRNA) [3,4]. In addition, mRNA-like RNAs also exist, as do long non-coding RNAs without poly-A tails [5]. These different non-coding RNAs play vital roles in transcriptional and post-transcriptional regulation through specific spatial and temporal expression (Figure 1) [6].

## 2. Biogenesis and Mode of Action

The emergence of non-coding RNAs has enriched the knowledge of the regulation of genetic expression in plants. These RNAs can enhance the adaptability and resistance of plants to environmental changes without altering the expression of genetic information [7,8].

### 2.1. Biosynthesis and Action Mechanism of microRNAs

**Figure 2:**
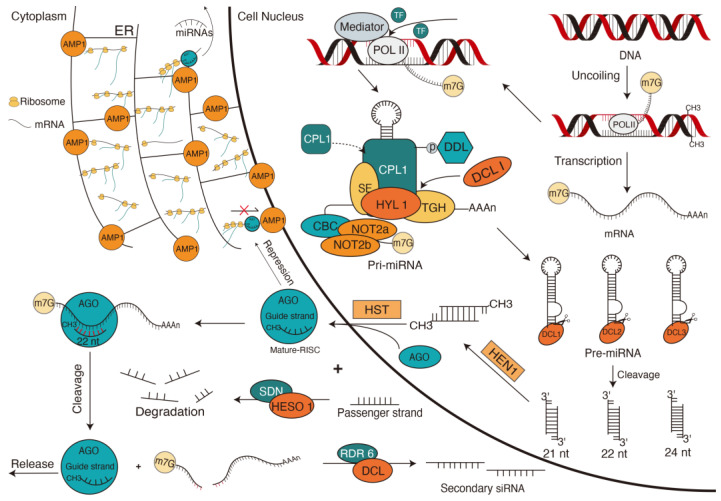
The biosynthesis and mechanism of micro RNAs (miRNAs). Many factors, such as transcription factors, mediators, and other protein complexes, affect the transcription of miRNA genes. Transcription factors (TFs) mediate POLII catalysis to unravel the miRNA gene (MIR). MIR is transcribed into stable pri-miRNA with stem-loop structure under the catalysis of DDL and CBC. TGH, SE, HYL1/DRB1, CPL1, and DCL proteins form a complex (D-body). DCL protein excises redundant sequences at the 5′ and 3′ terminals of the pri-miRNA stem base to form pre-miRNA. The DCL protein continues to cleave upward along the stem base of the pre-miRNA to form two complementary short double-stranded RNAs (dsRNAs). DsRNAs are methylated by HEN1 dsRNA terminal nucleotides and transported to the cytoplasm by HST. In the cytoplasm, SDN and HESO1 protein degrade one strand of dsRNA, while the other strand binds to AGO in RISC to form mature RISC. The fully functional RISC binds to the mRNA target sites under the guidance of miRNA and uses endonuclease activity to cleave the target mRNA. RISC can also prevent ribosome movement or promote ribosome shedding by binding mRNA to AMP1 on the endoplasmic reticulum. The RNA of the sheared mRNA fragment is degraded by an exonuclease and forms secondary small RNA. AMP1, ALTERED MERISTEM PROGRAM 1; CBC, nuclear cap-binding complex; CPL1, C-TERMINAL DOMAIN PHOSPHATASE-LIKE1; DCL, Dicer-like Protein; DDL, the phosphothreonine binding forkhead-associated domain protein DAWDLE; ER, endoplasmic reticulum; HESO1, the non-canonical poly (A) polymerase HEN1 SUPPRESSOR1; HYL1, HYPONASTIC LEAVES 1; m7G, a modification that a methyl group is added to the seventh N position of guanine (G) of mRNA; NOT2a/b, homologous proteins of the animal CCR4-NOT complex component NOT; POLII, RNA polymerase II; RDR6, RNA-dependent RNA polymerase 6; SDN, SMALL RNA DEGRADING NUCLEASE; SE, the zinc-finger protein SERRATE; TF, transcription factors; TGH, G-patch domain protein TOUGH.

MiRNAs are single-stranded molecules 20–24 nt in length that are commonly found in plants. MiRNAs are rigorous in terms of the proteins required for their biogenesis, the composition of the argonaute complex, and the gene regulation model [9]. The synthesis of miRNA can be divided into pri-miRNA, pre-miRNA, and miRNA steps (Figure 2) [10].

The miRNA gene (MIR) located in the intergenic region is transcribed by RNA polymerase II (POLII) to form a pri-miRNA in the nucleus [11]. Partial reverse-repeated base sequences will form an imperfect folded structure through pairing under the catalysis of RNA-binding protein DAWDLE (DDL) [12]. The stable pri-miRNA with a stem–loop structure is then formed under the catalysis of DDL and the nuclear cap-binding complex (CBC), which is formed by cap-binding proteins CBP20 and CBP80 [13]. The expression of MIR genes depends on the recruitment of the POLII through the interaction between transcriptional activators and the transcriptional coactivator mediator [14]. Experiments prove that at least 21 cis-acting elements, including the Goldberg–Hogness box (TATA-box), are overexpressed during transcription of the MIR gene, suggesting that the transcription process of the MIR gene is also regulated by a variety of trans-acting factors [13].

The dicing (D-bodies), which are a combination of the G-patch domain protein TOUGH (TGH), the zinc-finger protein SERRATE (SE) [15], HYPONASTIC LEAVES1 (HYL1/DRB1), C-terminal domain phosphatase-like1 (CPL1), and Dicer-like (DCL) proteins, will excise redundant sequences of 5′ and 3′ ends at the stem base of pri-miRNA to form pre-miRNA [16]. Although pre-miRNA exists for only a short time, this step plays an important role in ensuring accurate cleavage by the DCL protein in the subsequent steps. Studies have shown that DDL participates in the DCL1 complex by forming a fork-related (FHA) domain with a seven-strand β-sandwich structure at its carboxyl end. The residue of this domain is specifically recognized by the conserved phosphate-threonine binding cleft [17]. TGH binds to the region of single-stranded RNA, SE protein binds to pri-miRNA, and HYL1/DRB1 binds to the junction of single- and double-stranded RNA. TGH interacts with the transcription factor TATA-box binding protein 2 to enhance the activity of DCL1 in pri-miRNA processing, while also affecting the ability of HYL1 to bind to RNA. SE can maintain the low phosphorylation state of HYL1 by recruiting CPL1 and working with HYL1, thereby improving the cleavage accuracy of pri-miRNA by DCL1. The association between transcription mechanisms and components of miRNA processing suggests that D-bodies are determinants of the recruitment and maintenance of the active structure of the DCL protein and that pri-miRNA processing may be synergistic [13].

The pre-miRNA stem-loops are sheared by DCL protein along the base at 21 nt intervals to form double-stranded RNA (dsRNA) consisting of a miRNA* chain and a miRNA chain with 2-nucleotide 3′ overhangs [18]. A pre-miRNA can be recognized and cleaved by multiple DCLs, and different DCL family members produce different lengths of miRNAs. DCL1 and DCL4 produce 21 nt miRNAs, while DCL2 and DCL3 produce 22 nt and 24 nt longs miRNAs, respectively [19]. The length of the miRNA depends on the intramolecular spacing of the RNase III active site in the DCL protein and the 3′ overhang of the binding site in the PAZ domain [13].

The 3′ terminal nucleotides of miRNA/miRNA* (guide strand/passenger strand) duplex are methylated by a methyltransferase HEN1 to prevent uridylation and degradation. The miRNA/miRNA* duplex is then transported to the cytoplasm by HASTY (HST), where it can associate with an RNA-induced silencing complex (RISC) containing ARGONAUTE 1 (AGO1) proteins. These proteins regulate gene silencing by mRNA cleavage and translational repression [20]. The 5′ terminus of the guide strand is located at the less stably base-pairing end of the miRNA/miRNA* duplex. Due to this feature, the guide strand is recognized by the dissociated AGO protein and captured to form RISC [21,22] (Figure 2). Experiments have shown a reduction in only a small amount of miRNA accumulation in HST-deficient mutant plants, suggesting the existence of another HST-independent miRNA export pattern [23].

The other passenger chain is degraded by the SMALL RNA NUCLEASE protein (SDN) and the non-canonical poly (A) polymerase HEN1 SUPPRESSOR1 protein (HESO1) [24]. SDN1 has 3′-5′ exonuclease activity, is inhibited by 3′ oligouridylation, and prefers to cut short single strands. HESO1 can add a 3′ oligouridylate tail to unmethylated miRNAs, thereby marking them for degradation, and the activity of HESO1 is entirely inhibited by 2′-O-methylation of its substrates. Plant miRNAs possess a 2′-O-methyl group on each nucleotide’s ribose residue, which protects them from uridylation by HESO1. However, recent studies indicate that nucleotidyl transferase HESO1 can uridylate unmethylated miRNAs in *Arabidopsis thaliana* (Arabidopsis). Therefore, in addition to the SDN1 and HESO1 enzymes, other enzymes involved in the 3′ to 5′ degradation of uridylated miRNAs may contribute to the degradation of the passenger strand [25].

An example of a process regulated by miRNAs is flower development, which can be divided into three stages: flower meristem formation, flower organ differentiation, and sex differentiation [26]. At each stage of flower development, miRNAs regulate the expression of flowering genes at post-transcriptional and translational levels.

When the flowering genes are sufficiently expressed in the plant, the shoot apical meristem forms the floral meristem [26]. During this process, AGO10 and MIR165/166 will interact to specifically release CLASS III HOMEODOMAIN LEUCINE ZIPPER (HD-ZIP III) gene expression and maintain proper apical meristem development [27]. The floral meristem then forms floral organ primordia under the influence of various miRNAs that regulate the expression of floral organ recognition genes. This is a crucial step in flower development, as it determines the boundaries between various flower organs, and miRNAs play a key role in forming different flower organs. MiR164 affects sepal and leaf formation by regulating the NAC domain family of transcription factors (TFs) containing Cuc1 and Cuc2 genes [28]. Experiments showed that miR164-deficient functional mutants formed additional petals. Overexpression of miR164 in *Solanum lycopersicum* resulted in dysplastic floral organ and leaf development. Besides that, miR164 was also expressed at the highest level in crop plants, including *Solanum lycopersicum*, *Vitis vinifera* L. and *Citrus sinensis* [29]. After the organs are fully differentiated, the floral organs will further develop and undergo sexual differentiation, producing stamens and pistils [26].

Compared with post-transcriptional regulation, translational regulation seems more widespread in plants. In the absence of significant changes in mRNA quantity, miRNA regulation on gene expression is insensitive to AGO1 cleavage inhibition and results in significant changes in protein quality. This suggests that after RISC binds to a target mRNA, more mRNAs enter the endoplasmic reticulum (ER) and bind to ribosomes. ALTERED MERISTEM PROGRAM 1 (AMP1) (Figure 2) is present in the ER and is involved in miRNA-mediated translation repression processes at the protein level, such as inhibiting translation initiation, hindering ribosome motility, or promoting ribosome shedding. This also demonstrates that the functional role of the ER in the miRNA-mediated translational inhibition [30].

### 2.2. Biosynthesis and Action Mechanism of siRNAs

**Figure 3:**
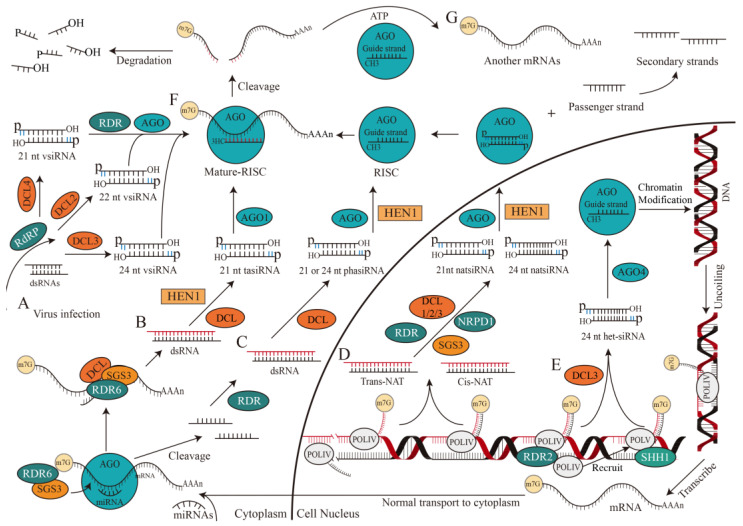
The biogenesis and mechanism of small interfering RNAs (siRNAs). The biogenesis and mechanism of siRNAs begin with the cleavage of nucleic acid, RISC formation and product release. The exogenous dsRNAs from virus infection in the cytoplasm are cleaved by DCL to form vsiRNA and combined with a member of the AGO protein family to form a mature RISC (**A**). In the cytoplasm, the cleavage of the transcript by miRNA and AGO1 recruits SGS3 and RDR6. The RDR6 replicated SGS3 catalyzes transcript to form dsRNA, which will be cleaved by DCL and methylated by HEN1 to form a 21 nt tasiRNA that will bind to AGO1 to form a mature complex RISC (**B**). Under the co-catalysis of RDR, DCL and HEN1, the nucleic acid fragments produced by the miRNA-mediated mRNA cleavage process are transformed into 21 or 24 nt phasiRNA (**C**). Catalyzed by RDR, SGS3 and NRPD1, DCL1/2/3 cleaves NATs to produce 21 and 24 nt NatsiRNAs (**D**). After catalytic and cleavage by POLIV, RDR2 and DCL, 24 nt het-siRNAs load into AGO4 protein, which induced inhibitory chromatin modification and transcription gene silencing. In addition, SHH1 protein can recruit POLIV to synthesize siRNA at the transcription site of POLV (**E**). Mature RISC recognizes complementary target RNAs and cleaves them at positions between t10 and t11 (**F**). In some cases, highly purified RISCs cannot cleave their target mRNAs in a multi-conversion manner, suggesting the presence of external factors possibly driven by ATP hydrolysis (**G**). AGO, argonaute protein family; ATP, adenosine triphosphate; DCL, Dicer-like protein family; dsRNA, double-stranded RNA; HEN1, methyltransferase; het-siRNA, heterochromatic siRNA; m7G, a modification that a methyl group is added to the seventh N position of guanine (G) of mRNA; NATs, natural antisense transcripts; natsiRNA, natural antisense siRNA; NRPD1, DNA-directed RNA polymerase IV subunit 1; phasiRNA, phased small interfering RNA; POLIV, RNA polymerase IV; POLV, RNA polymerase V; RDR, RNA-dependent RNA polymerase; RdRP, RNA-dependent RNA polymerases; RISC, RNA-induced silencing complex; SGS3, SUPPRESSOR OF GENE SILENCING 3; SHH1, SAWADEE HOMEODOMAIN HOMOLOGUE 1; tasiRNA, trans-acting siRNA.

The origin of siRNAs (~20–30 nt), is derived directly from viruses, transposons, or transgene triggers and is considered exogenous and endogenous (Figure 3) [22,24]. Exogenous siRNA is derived from viral RNA in plants after virus infection [31]. RNA-dependent RNA polymerases (RdRP) may recognize and use these abnormal RNAs as templates to synthesize antisense RNAs and form dsRNAs. DCL2/3/4 then processes these dsRNAs to generate 22, 24, and 21 nt primary vsiRNAs, which are subsequently amplified by RNA-dependent RNA polymerase (RDR) and loaded into AGO to form RISCs (Figure 3A) [32].

Evidence supports that endogenous siRNAs include phased small interfering RNAs (phasiRNAs), heterochromatic siRNAs (het-siRNAs), trans-acting siRNAs (tasiRNAs), and natural antisense siRNAs (natsiRNAs), which could be originated from endogenous sequences, including transposons, repetitive elements, or tandem repeats in plants and aim to cleave target RNAs [33,34,35,36]. TasiRNAs, one of the products of small RNA interactions, are generated when a transcript is cleaved by a complex constituted by miRNA and AGO1. This process will recruit SUPPRESSOR OF GENE SILENCING 3 (SGS3) and RDR6 to duplicate the transcript and generate dsRNAs through RDR6 with the assistance of SGS3 [6,37]. The dsRNAs are then cleaved and methylated by DCL and HEN1 to be processed into 21 nt tasiRNA, which combines with AGO1 to cleave the target RNA (Figure 3B) [38].

The biogenesis of phasiRNA is related to miRNA, which mediates the cleavage of AGO protein from the 3′ end to single-stranded RNA precursor, which is converted to dsRNA by RDR protein [39], and then processed into 21 or 24 nt phasiRNA by DCL protein. When mRNA contains two miRNA target sites, only one site is cleaved (usually at the 3′ terminal site) and the mRNA is then cleaved continuously by DCL to produce a 21 or 24 nt phasiRNA (Figure 3C) [40].

NatsiRNAs, on the other hand, are produced by natural antisense transcripts (NATs) through the cleavage of DCL1/2/3 in the presence of RDR [41], SGS3, and DNA-directed RNA polymerase IV subunit 1 (NRPD1). NATs are formed by two complementary RNA strands that are separately transcribed and can be divided into cis-NAT and trans-NAT, depending on their genomic source. Cis-NAT is transcribed from the same genomic locus, and forms a fully complementary dsRNA. While trans-NAT constitutes highly complementary dsRNAs, transcribed from two distant genomic sites. NatsiRNAs can be further divided into 21 and 24 nt cis-natsiRNAs or trans-natsiRNAs, depending on the origin of NATs [42] (Figure 3D).

According to current knowledge of the RNA-directed DNA methylation (RdDM) pathway, the biogenesis of het-siRNA is dependent on RNA polymerase IV (POLIV) [43], RDR2 [44], and DCL3 (mainly) [45]. Het-siRNA is commonly loaded into AGO4 and leads to inhibitory chromatin modification and transcriptional gene silencing, which is crucial for transposition element silencing, stress response, and genome stability [46]. The SAWADEE HOMEODOMAIN HOMOLOGUE 1 (SHH1) protein interacts with chromatin at the RNA polymerase V transcription site, recruiting POLIV to promote siRNA biosynthesis (Figure 3E) [47]. Additionally, AGO4 binds to 24 nt het-siRNA and facilitates the classical RdDM pathway in a non-redundant manner [48,49].

The synthesis of siRNAs can be divided into the following steps. (1) Various DCL enzymes [50] catalyze dsRNAs with RNase family ribonuclease activity to generate two siRNA double strands with 5′ phosphoric and 3′ hydroxyl groups, each strand contains 3′ two-nucleotide overhangs (represented by blue lines). (2) The 3′ end of guide and passenger strand of siRNA are methylated by a reaction catalyzed by HEN1 [51]. (3) AGO protein [52] binds to guide strand of siRNA to form the argonaute-small RNA complex—RISC [53], and the passenger strand is then released from RISC, cleaved and degraded. (4) Mature RISCs assemble in the cytoplasm and perform biological functions (Figure 3).

Studies have shown that more than 70% of the siRNA within a plant cell are 24 nt heterochromatic siRNAs which do not cleave target mRNA nor act at the post-transcriptional level [22]. However, other functional siRNAs are more inclined to cleave target mRNA at transcription and post-transcriptional levels, thereby leading to gene silencing in gene expression regulation. This gene silencing is also called RNAi [54]. In the canonical RNAi pathway, a few dsRNA molecules can induce strong RNAi reactions [55]. RNA-induced silencing complex guided by siRNA (si-RISC) recognizes target mRNA through base-complementary pairing. It is worth noting that full-sequence complementarity is not always necessary for binding, as imperfect annealing and incomplete base-complementary pairing are allowed. The si-RISC cleaves mRNA into fragments with the PIWI domain, which possesses catalytic activity in the AGO protein [22,54,56]. The reorganization and cleavage of RISC are precise: si-RISC effectively identifies and cuts target RNAs with broad complementarity, specifically at positions between t10 and t11 (Figure 3F) [57]. Exonucleases will further degrade part of the small fragments, and the other part will become the precursor of secondary siRNA [58]. However, in some cases, highly purified RISCs cannot cleave their target mRNAs in a multi-conversion manner, suggesting the presence of external factors that promote RISC and product release, possibly driven by ATP hydrolysis (Figure 3G) [59]. Studies have shown whether the secondary siRNAs are produced by tasiRNAs through a unique miRNA-dependent mechanism or by the interaction between lincRNAs and miRNAs [60,61], nearly all secondary siRNAs correspond to antisense strands of primary dsRNA-targeted mRNAs, which contain 5′ di- or triphosphate groups.

### 2.3. Biosynthesis and Action Mechanism of circRNAs

The circRNAs are another type of endogenous non-coding RNAs with a covalently closed-loop structure [62]. As the first detected circular RNAs were viroids, circRNAs have ever been thought of as faulty cuts or by-products of the transcription [63]. However, with advances in high-throughput sequencing technologies, an increasing number of circRNAs have been shown to regulate transcription, alternative splicing, and other biological processes. The unique closed-loop structure makes circRNAs more stable than linear non-coding RNAs and less easily degraded by ribonuclease. Studies have shown that circRNAs have a much longer half-life than linear RNAs [64]. Therefore, circRNAs can remain in cells for a longer duration and continuously regulate biological processes. This is of great significance in plants’ adaptation to and co-evolution with the environment.

**Figure 4:**
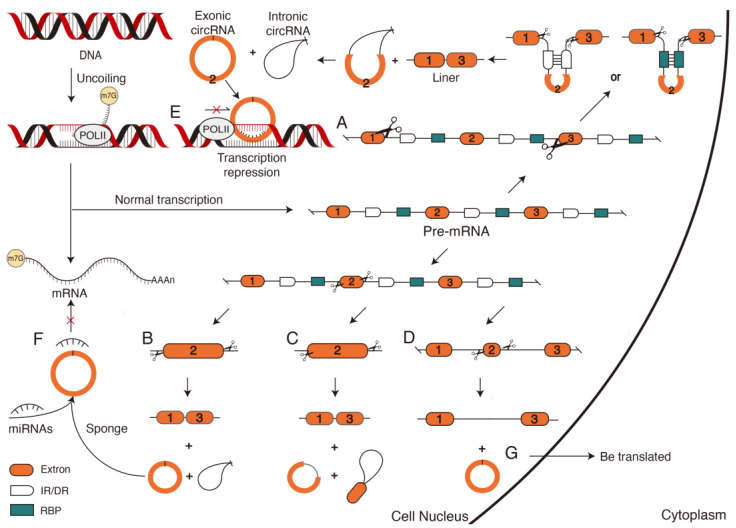
The biosynthesis and mechanism of circular RNAs (circRNAs). Different splicing of pre-mRNA produces different types and functions of circRNA. Exonic and intronic circRNAs are formed after pre-mRNA is cleaved at exon ends and flanking sequences containing long introns (multiple RBPS and IR or DR) (**A**). Cleavage of both ends of an exon at the pre-mRNA will result in the formation of linear RNA, exonic circRNA, and intronic circRNA (**B**). Exon–intron circRNA and intron–exon circRNA are generated when the cleavage initiation or termination site occurs within an exon of the pre-mRNA (**C**). When the exon of pre-mRNA is short, the longer linear RNA and exon circular RNA can be formed from the remaining sequences (**D**). The generated circRNA types have different functions; for example, exonic circRNAs can directly hybridize with target DNA to form a DNA:RNA structure, preventing the linear RNA formed by transcription from binding to DNA, resulting in transcriptional pause (**E**). CircRNAs can also act as miRNA sponges to regulate gene expression indirectly (**F**). CircRNAs have certain coding abilities and can also be translated into proteins (**G**). DR, direct repeat sequence; IR, reverse repeat sequence; RBP, RNA binding protein.

Unlike siRNAs and miRNAs, which are formed by linear splicing, circRNAs are formed by covalently connecting the downstream 3′ donor splice site to the upstream 5′ receptor splice site via reverse splicing mediated by complementary base pairing [65]. The formation of circRNAs relies on cleavage sites provided by complementary or reverse complementary, direct repeat sequence (DR) or reverse repeat sequence (IR) in introns and splicing action of the spliceosome (Figure 4A). This results in the generation of circRNAs and exon-skipping linear RNAs, which will either be directly degraded or translated into a truncated protein different from the native protein [66]. Therefore, according to different splicing sites of reverse cleavage, the formation of circRNAs can be divided into two models: the reverse splicing model and the intermediate lasso model [67]. The formed circRNAs are divided into exonic circRNA, intronic circRNA (Figure 4B), exon–intron circRNA, intron–exon circRNA (Figure 4C), UTR circRNA, UTR–exon circRNA, UTR–intron circRNA, intergenic circRNA, intergenic–genic circRNA, and across circRNA [68]. The remaining sequences will form linear RNA, and it will form a longer linear RNA when an exon is short (Figure 4D). However, recent studies have verified that at least 30–40 nt lengths of reverse complementary repeats (RCRs) in introns are sufficient to form circRNAs [69]. The 103 nt RCR can even improve circRNA biogenesis efficiency by a factor of 1.3 [70], suggesting that the flanking intron sequences are unnecessary for circRNA biogenesis. The stable and unique closed-loop structure generated by various pathways enables circRNAs to function in cells.

Circular RNAs in cells regulate gene expression at the transcriptional level in two ways. The first inhibitory regulation prevents binding between transcriptionally formed linear RNA and DNA, resulting in the strong binding of the circRNA to the target DNA site to form a hybrid DNA:RNA structure [63], which directly suspends transcription (Figure 4E). For example, circSEP3 transcribed from the Arabidopsis SEPALLATA3 gene binds much more strongly to homologous DNA than to linear RNAs of the same sequence. Therefore, a circRNA:DNA structure is formed to suspend transcription and form alternatively spliced SEP3 mRNA with exon skipping [71]. Another type of activated regulation is indirect regulation through the competitive binding of circRNAs to regulators such as miRNAs (Figure 4F) to reduce target mRNA cleavage and maintain a normal transcription [72].

High-throughput sequencing and bioinformatics analysis have revealed the presence of circular RNAs in mature fruits. Many different circRNAs are specifically expressed at different fruit ripening stages [73]. CircRNAs are also explicitly expressed in the fruit ripening stage of different plants, such as Arabidopsis, *Oryza sativa*. Their sequence similarities indicate that the expression of circRNAs is ubiquitous in cells at different stages and is conserved as specific expression patterns in different species [72].

CircRNAs are also induced under various abiotic and biotic stresses, such as low temperature, high temperature or nutrient deficiency. The first circRNA produced under biotic stress was discovered in pathogen-infected Arabidopsis plants [74]. Subsequently, at low temperatures, 475 differentially expressed circRNAs were identified in grape leaves. CircRNAs enhanced cold tolerance in Arabidopsis by regulating the expression of the *CSD2*, *PRXCA*, *PME41*, *LOX3,* and *WRKY48* genes [75]. CircRNAs are also acting as signaling molecules to affect the process of cellular communication and as biomarkers for studying the physiological activities and regulatory patterns of plant cell responses to environmental stress (Figure 4F) [63].

### 2.4. Biosynthesis and Action Mechanism of lincRNAs

The lincRNAs are a class of non-coding RNAs with a length of more than 200 nt. Their sequences have low conservation between different species and are widely involved in plant growth and stress responses [76,77]. Their biosynthesis is similar to mRNA synthesis and requires POLII to initiate catalytic transcription with the help of TFs. Some lincRNAs can also be transcribed by RNA polymerase III, and a few lincRNAs in plants are produced by the plant-specific POLIV and V [77].

**Figure 5:**
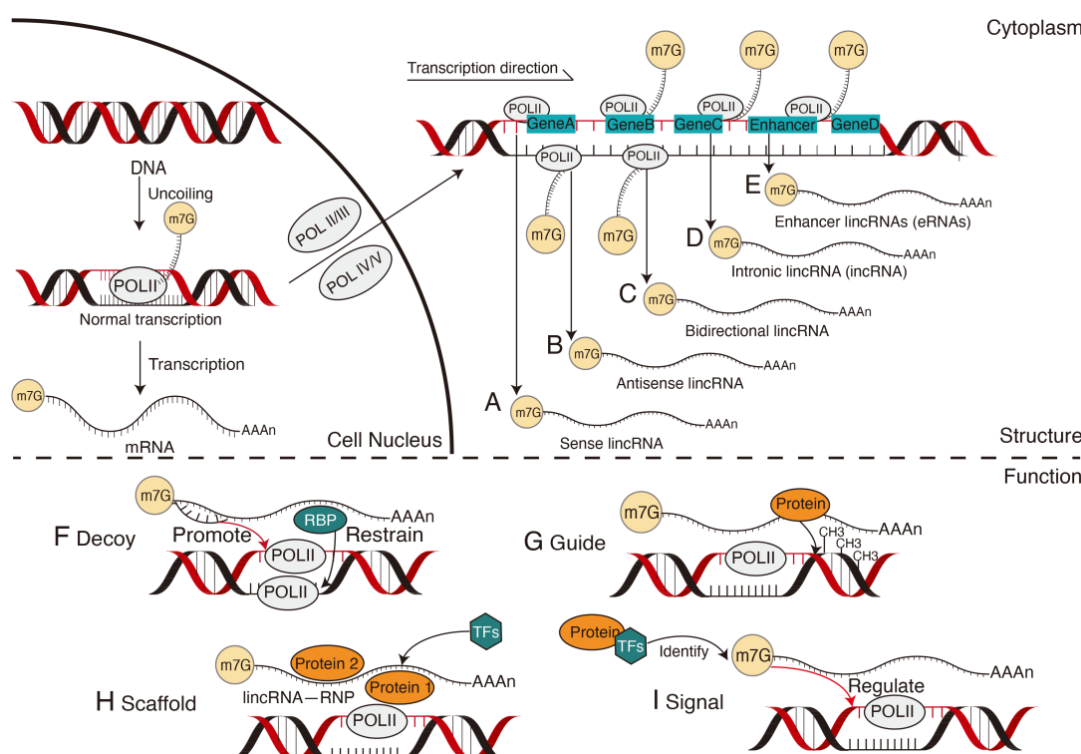
The biosynthesis and mechanism of long intergenic non-coding RNAs (lincRNAs). The synthesis of lincRNAs is similar to mRNA, in which DNA is transcribed into precursor RNA sequences catalyzed by TFs and RNA polymerase and transported to the cytoplasm for modification to perform different functions. LincRNAs can be classified according to different nuclear transcription sites: sense, antisense, bidirectional, intronic, and enhancer lincRNAs (**A**–**E**). The lincRNAs have several functions, such as decoy (**F**), where lincRNAs bind RNA-binding proteins (RBPs) to regulate gene expression. The lincRNAs can be a guide (**G**) to regulate gene expression by recruiting proteins to bind directly or form complexes with proteins that then bind to target gene-specific sites. The lincRNAs can act as a scaffold (**H**) by constituting the assembly sites of biological macromolecules and the necessary components for their function. Moreover, lincRNA can act as a molecular signal (**I**) to regulate gene expression at a specific time. m7G, a modification that a methyl group is added to the seventh N position of guanine (G) of mRNA; POLII, RNA polymerase II; POLIII, RNA polymerase III; POLIV, RNA polymerase IV; POLV, RNA polymerase V; TF, transcription factor.

Unlike mRNA transcribed from the corresponding gene, lincRNA transcription sites are variable. Therefore, according to the position of the lincRNAs in the genome relative to adjacent protein-coding genes, lincRNAs can be divided into sense lincRNAs (Figure 5A) and antisense lincRNAs (Figure 5B), which are the same or complementary to one (or more) exons of protein-coding genes at the overlapping position. Bidirectional lincRNAs (Figure 5C) [78] are expressed simultaneously with encoded transcripts on opposite strands. Intronic lincRNA (incRNA, Figure 5D) are derived from introns of protein-coding genes; large intergenic lincRNA [77] and enhancer lincRNAs (eRNAs, Figure 5E) are derived from the enhancer region of protein-coding genes. The transcriptional localization of lincRNA at different genomic locations suggests the following mechanism by which lincRNA regulates the gene expression [79].

1. Decoys (Figure 5F): Transcriptional lincRNAs regulate gene expression by binding RBPs; this is the main regulation mechanism of lincRNA. RBPs bound by lincRNA could be TFs, chromatin modifiers, or other regulatory factors, while lincRNAs have no other function. LincRNAs conforming to this regulatory mechanism not only bind to RBPs required for standard transcription of mRNA but also play a negative regulatory role in the promoter region by forming complexes with RBPs to prevent normal transcription of mRNA. Deletion of lincRNA will result in a rescued phenotype [80]. However, some lincRNAs act as decoys in plants but increase gene expression. Those lincRNAs are usually intergenic, contain multiple coding genes, have long sequences like mRNA, and post-transcriptionally have multiple miRNA-binding sites. Therefore, these lincRNAs are also called endogenous competing RNAs. They act as “sponges” to competitively bind miRNAs and indirectly regulate the expression of miRNA target genes by removing their inhibitory effect [81]. This mode of action is found in the stress response and fruit development. As miRNA targets, IPS1, the first lincRNA as decoys, has a region partially complementary to miR399 and sequesters miR399 to reduce the availability of free miR399 for degradation and inhibiting complete silencing of PHO2 mRNA [82]. LNC1 and LNC2 indirectly affect the expression of SPL9 and MYB114 genes, which are initial targets of miRNA, thereby controlling anthocyanin biosynthesis during fruit ripening [83].
2. Guides (Figure 5G): LincRNA regulates gene expression by recruiting proteins to bind directly or by forming complexes with proteins and then binding target gene-specific sites, which can be a neighboring gene or a remote gene [80]. Predicting this regulation from the sequence of lincRNA is difficult, but lincRNA allows local changes in chromosome structure to have local effects (cis) and distant effects (trans). Regardless of whether it is cis or trans, the principle is the same: gene expression is controlled by changing the structure state of chromosomes to transmit regulatory information to control gene expression, resulting in epigenome variations. In fact, lincRNAs such as AIR and eRNAs, transcribed from promoters or enhancers, regulate gene expression in cis. For example, COLD ASSISTED INTRONIC NONCODING RNA (COLDAIR) and COOL ASSISTED INTRONIC NONCODING RNA (COOLAIR) in Arabidopsis recruit the protein complex Poly-comb Repressive Complex 2 (PRC2) to the FLOWERING LOCUS C (FLC). H3K27 methyltransferase modification changes the chromatin structure of FLC under the action of PRC2, inhibits the expression of FLC, and regulates flowering time [84]. This epigenetic transcriptional silencing profoundly affects the spatiotemporal specific expression of plant genes under stress.
3. Scaffolds (Figure 5H): Scaffold complexes composed of proteins have traditionally been considered as assembly sites and essential components of the biological macromolecule [79]. Recent studies have shown that lincRNAs can also serve as central platforms for the assembly of related molecules, critical for precise control of intermolecular interactions and specificity of signal transduction [85]. Using lincRNA as a scaffold, different or identical proteins can aggregate to form a ribonucleic protein complex lincRNA-RNP. LincRNA-RNPs affect the target gene histone modifications in the cis (or trans) group by binding TFs to activate or inhibit gene expression by recruiting chromatin modification enzymes [79].
4. Signals (Figure 5I): Gene expression is spatiotemporally specific. Plants express different genes at different times and places under different environmental stimuli. Transcription of lincRNA also occurs at specific times and locations as lincRNA synthesis is closely related to the mRNA transcription [86]. Therefore, lincRNAs can act as molecular signals. When lincRNAs are recognized by TFs, the proteins and other factors involved in the regulatory process will initiate positive or negative regulation of genes to regulate gene expression, thereby simplifying the identification of the chromatin state of regulatory elements. Moreover, harnessing RNA as a signaling molecule for gene regulation can expedite the development of stress resistance in plants, allowing them to swiftly adapt to natural pressures and thrive in their surroundings with enhanced efficiency. This regulation is independent of the recognition and catalysis of transcriptionally produced proteins. For example, cold causes epigenetic silencing of the FLC [87], resulting in the activation of the COLDAIR promoter in the first intron of the FLC gene. COLDAIR is transcribed along the sense strand of the FLC intron and acts as a signaling molecule to recruit PRC2 to methylate FLC histone, repress FLC expression, and provide plants with the flowering ability [88].

## 3. Conclusions and Future Perspectives

With the discovery and identification of many non-coding RNAs, the identification and understanding of non-coding RNAs are growing. The discovery and study of non-coding RNA explain the high number of repetitive sequences in plant genomes, and they explain the phenomenon that more than three-quarters of transcripts do not encode. This also confirms that non-coding RNAs play an irreplaceable key role in regulating gene expression, laying the foundation for plant adaptation to the environment and coordinated evolution with the environment.

Here, we have reviewed the regulation of gene expression by non-coding RNAs at different stages of plant growth and development. The regulation of gene expression by non-coding RNA is multi-level, multi-factored, and multi-pathed. Meanwhile, the regulation of gene expression by different non-coding RNAs is also an interactive and co-regulated process rather than an independent process. Plant non-coding RNAs, by far, are poorly studied compared to those of animals and lack sufficient experimental and functional validation. The coding ability of non-coding RNAs and their roles in plant growth and development (such as leaf senescence, flower development, fruit ripening and response to biotic and abiotic stresses) may be major research topics for non-coding RNAs in plants. These topics still require extensive whole-transcriptome sequencing and functional validation. In addition, the differences between plant and animal non-coding RNAs will also be an interesting topic worthy of further exploration.

## Data Availability

Not applicable.
